# Sepsis and ARDS: The Dark Side of Histones

**DOI:** 10.1155/2015/205054

**Published:** 2015-11-01

**Authors:** Zhiheng Xu, Yongbo Huang, Pu Mao, Jianrong Zhang, Yimin Li

**Affiliations:** ^1^State Key Laboratory of Respiratory Diseases, The First Affiliated Hospital of Guangzhou Medical University, Guangzhou 510120, China; ^2^Infection Control Department, The First Affiliated Hospital of Guangzhou Medical University, Guangzhou 510120, China; ^3^Department of Thoracic Surgery, The First Affiliated Hospital of Guangzhou Medical University, Guangzhou 510120, China

## Abstract

Despite advances in management over the last several decades, sepsis and acute respiratory distress syndrome (ARDS) still remain major clinical challenges and the leading causes of death for patients in intensive care units (ICUs) due to insufficient understanding of the pathophysiological mechanisms of these diseases. However, recent studies have shown that histones, also known as chromatin-basic structure proteins, could be released into the extracellular space during severe stress and physical challenges to the body (e.g., sepsis and ARDS). Due to their cytotoxic and proinflammatory effects, extracellular histones can lead to excessive and overwhelming cell damage and death, thus contributing to the pathogenesis of both sepsis and ARDS. In addition, antihistone-based treatments (e.g., neutralizing antibodies, activated protein C, and heparin) have shown protective effects and have significantly improved the outcomes of mice suffering from sepsis and ARDS. Here, we review researches related to the pathological role of histone in context of sepsis and ARDS and evaluate the potential value of histones as biomarkers and therapeutic targets of these diseases.

## 1. Introduction

Over the last several decades, severe sepsis and acute respiratory distress syndrome (ARDS) have been the most common causes of mortality in critically ill patients [1–3]. During these years, a growing number of advanced interventions and strategies have been applied to critically ill patients. Pharmacological interventions, including antithrombin III [4], tifacogin [5], vasoactive drugs [6, 7], and activated protein C [8], have been proven to be helpful. Moreover, the strategies of mechanical ventilation are of vital importance. With an increasing use of noninvasive positive-pressure ventilation, a reduction in tidal volume, and an increase in applied positive end-expiratory pressure [9], the mortality of critically ill patients with sepsis and ARDS has gradually decreased over the last decade [9, 10]. However, the mortality rates still remain unacceptably high, with a 20 to 30% mortality rate from sepsis [11] and a mortality rate greater than 40% from ARDS [12].

Despite advanced developments in life support management (e.g., ventilators, dialysis, and extracorporeal membrane oxygenation), these interventions are not specific for blocking or targeting the pathogenic processes of these diseases. Therefore, a comprehensive treatment for critical illness should include not only alleviating the pain but also targeting the underlying pathological mechanism. However, the underlying mechanisms of ARDS and sepsis remain largely unknown. Sepsis and ARDS result from complex events such as infections, trauma, burning, and acid aspiration [13], which trigger innate and adaptive immune responses. The complexity of these processes involves complement system activation, neutrophil infiltration, vascular endothelial system damage, coagulation cascades promotion, and barrier dysfunction [14, 15]. Therefore, for a better understanding of the pathophysiological process of sepsis and ARDS, additional molecular mechanisms need to be explored.

It appears to be widely accepted that investigating the targets that are abnormally expressed in critically ill patients and in animal models holds promise for identifying new underlying molecular mechanisms. Recently, it has been reported that histones, as basic and important structural elements in nuclear chromatin and the regulation of gene transcription, can be released passively into the extracellular space when cells undergo severe injury, giving rise to immunostimulatory and cytotoxic effects on both sepsis [16, 17] and ARDS [18, 19].

Before they are released into the extracellular space, histones are the major proteins of chromosomes found in eukaryotic cell nuclei and are highly conserved across species. There are five families of histones known to date: H2A, H2B, H3, and H4, which are known as “core histones,” and histone H1 and its homolog H5, which are known as the linker histones [20–22]. Histones are the basic structural elements in the nucleosome, which contains one H3/H4 tetramer and two H2A/H2B dimers, while H1 binds to nonnucleosomal DNA and facilitates numerous nucleosomes to form higher-order chromatin structures [20, 23]. Even though histones are extremely inert in the nucleus, they lead to significant pathogenic effects outside of the cells.

Mounting evidence from clinical and experimental data indicates that extracellular histones could act as new members of damage-associated molecular pattern molecules (DAMPs) [24–26]. The results from both patients and animal models have suggested that circulating histones play a crucial role in sepsis and ARDS and could serve as novel biomarkers as well as promising therapeutic targets [27, 28]. Therefore, a deeper understanding of the functions of extracellular histones may yield pivotal insights into the pathogenesis of sepsis and ARDS. In this review, we will focus on the pathogenic effects and clinical relevance of extracellular histones and hope to help set the stage for future studies.

## 2. The Source of Extracellular Histones

The source of extracellular histones is complicated. Histones are reported to be released from dying cells [29, 30]. During necrosis, accompanied by disruption of the cell plasma membrane, intracellular components are released into the extracellular space, and some (e.g., HMGB1, DNA, and histones) have the ability to activate innate immunity and cause more injury. Although apoptotic cells are in silent death without membrane disintegration [31], they are also thought to release histones by leaking from membrane blebs [32] and nucleosomes [33], which are produced by actin-myosin contractions during apoptosis.

In addition, the release of histones is also considered to be associated with neutrophil extracellular traps (NETs) [34]. NETs are formed by dying neutrophils that release DNA, histones, and granular proteins, such as neutrophil elastase and myeloperoxidase. In this way, the released histones play a predominant role in further inducing epithelial and endothelial cell death [35]. Therefore, extracellular histones can also be released by forming NETs. Another possible source of histones is large numbers of apoptotic and necrotic cells overwhelming the clearance ability of mononuclear phagocytes, thereby allowing histones to enter the circulatory system [36].

## 3. The Receptors of Extracellular Histones

Toll-like receptors, including toll-like receptors 2, 4, 9 (TLR2, TLR4, and TLR9), have been shown to be receptors of extracellular histones [25, 30, 37–39]. For example, histones promote plasma thrombin generation via TLR2 and TLR4 activation [39]. In addition, in the context of acute kidney injury, histones can induce leukocyte accumulation, renal inflammation, and microvascular leakage in TLR2/TLR4 dependent mechanism [30]. Moreover, extracellular histones are mediators of death in inflammatory injury and in chemical-induced cellular injury through TLR2 and TLR4 signaling [38]. Furthermore, endogenous histones mediate sterile inflammatory liver injury via TLR9 in mice [37]. However, Abrams and his colleagues [18] suggested that blockading TLR4 and TLR2 in trauma-associated lung injury models showed no protective effects, indicating that the activation of TLR2 and TLR4 may not be major pathway responses for histone toxicity. Collectively, in different disease models, extracellular histones may activate different toll-like receptors, including TLR2, TLR4, and TLR9, to mediate various pathogenic effects.

However, activated protein C (APC) and specific antibodies to histones can significantly reduce cytotoxicity and the mortality of septic mice by hydrolyzing or neutralizing histones, respectively [16, 18]. In addition, the protective effects of blockading TLR4 and TLR2 remain controversial. A report by Xu et al. [38] showed that TLR4 knock-out mice were protected from the fatal effects of histone infusion, and Ekaney et al. [17] demonstrated that blockading TLR4 decreased cellular cytotoxicity in endothelial cells. By contrast, Abrams and his colleagues [18] suggested that blockading TLR4 and TLR2 could not block a calcium influx when endothelial cells were treated with histones. These results indicate that TLR2 and TLR4 are receptors of histones. Blockading TLR2 and TLR4 may be protective; however, the exact mechanisms may differ in different disease models, and further investigation is needed.

## 4. Pathologic Roles of Extracellular Histones in Sepsis

Sepsis is a systemic inflammatory response to infection [40]. During the past two decades, it has remained an important clinical challenge in the intensive care unit (ICU) and one of the leading causes of death [41] due to an incomplete understanding of its pathophysiological mechanisms. Numerous studies in the field of sepsis have identified host response, innate immunity, coagulation abnormalities, and the balance between proinflammation and anti-inflammation as essential contributors to sepsis. Recently, Xu et al. [16] and Ekaney et al. [17] demonstrated that extracellular histones were major mediators in endotoxemia and septic shock through cytotoxicity, excessive inflammation, and coagulation dysfunction.

### 4.1. Cytotoxic Effects

High levels of extracellular histones are cytotoxic to both epithelial and endothelial cells [16, 18, 19, 35, 42]. Xu et al. [16] treated endothelial cells, specifically EA.hy926, with a mixture of purified mixed histones and five individual histones. They found that a mixture of histones was cytotoxic to these cells and the toxic effects were mainly due to histones H3 and H4. In addition, Abrams and his colleagues [18] demonstrated that sera from patients were toxic to cultured endothelial cells once histone levels exceeded 50 *μ*g/mL. Sera from sepsis patients directly induced histone-specific cardiomyocyte death, which further contributed to the development of cardiac injury, arrhythmias, and left ventricular dysfunction [43]. Interestingly, extracellular H1, but not H2A/H2B, H3, and H4, is neurotoxic and induces dramatic neuronal death [44].

However, the mechanism of the toxic effect of extracellular histones is not completely clear. It has been reported that positively charged histones could bind to negatively charged phospholipids in the plasma membrane [18, 45], leading to increased transmembrane conductance [46, 47], membrane disruption [18] and, finally, calcium influx [18, 48, 49]. Moreover, lymphocyte apoptosis induced by histones during sepsis is dependent on p38 phosphorylation and mitochondrial permeability transition [50]. Further studies have found that sera from survivors of septic shock were able to specifically induce dendritic cell (DC) apoptosis in a caspase-dependent pathway, and sera from nonsurvivors were able to induce DC-regulated necrosis, which could be abrogated by antihistone therapy [51].

### 4.2. Triggering and Promoting Inflammation in Sepsis

The innate immune system plays a crucial role in the pathophysiology of sepsis, which induces overwhelming systemic inflammation by releasing various inflammatory mediators in response to invading pathogens [14, 52]. In addition, histones could serve damage-associated molecular pattern molecules [53] involved in the aggravation of systemic inflammation. Recent studies have demonstrated that the release of histones contributes to the considerable production of sepsis-associated cytokines, such as TNF-*α*, IL-6, and IL-8, as well as IL-1*β*, and leads to cytokine storm [17, 54]. There are several reasons for this, detailed below.

First, histones could interact with TLR2 and TLR4 as ligand receptors and directly activate myeloid differentiation primary response gene88 (MyD88) to initiate inflammation [30, 38]. However, there may be some differences between histones and TLR9 interaction. TLR9 is an intracellular molecule that functions as a receptor of DNA [55, 56] and, therefore, histones bind to DNA and then enter the intracellular space to enhance the DNA-activated TLR9 signaling cascade [37].

Second, histones can activate monocyte-derived dendritic cells via the NLRP3 inflammasome to induce the production of IL-1*β* [45, 57, 58]. Lipopolysaccharide (LPS) pretreatment, followed by the addition of histones, showed significantly amplified production of IL-1*β* from the wild-type macrophages but not from NLRP3-defected macrophages, indicating that histones activated the NLRP3 inflammasome in macrophages to induce the release of IL-1*β* [59]. Activating the NLRP3 inflammasome with histones could promote the recruitment of neutrophils and the additional release of histones into the extracellular space, which establishes a vicious cycle that enhances inflammation [59, 60].

Third, histone-induced inflammation can be amplified by DNA and polyphosphate [37, 61]. Extracellular histones can enhance TLR9-mediated inflammation by interacting with DNA [37]. Moreover, polyphosphate amplifies H4-mediated inflammation in human umbilical vein endothelial cells specifically through interaction with the receptor for advanced glycation end products (RAGE) and P2Y1 [61].

Last, the charge itself may have proinflammatory effects [45]. Histones with highly positive charges are responsible for cytotoxicity and barrier dysfunction by charge-charge interaction [18]. In this regard, the geometry, topology, or density of the charge may determine the immune activity [45], but further investigations are needed.

### 4.3. Coagulation and Thrombosis in Sepsis

Sepsis is almost inevitably associated with the activation of blood coagulation (hypercoagulability) and systemic clotting with massive thrombin and fibrin formation, eventually resulting in the consumption of platelets and disseminated intravascular coagulation (DIC) [36, 62, 63]. Recent reports have suggested that extracellular histones triggered platelet aggregation and clotting both in vivo and in vitro [39, 64–67]. Therefore, by the consumption of platelets, histone-treated mice showed thrombocytopenia, prolonged prothrombin time, decreased fibrinogen, fibrin deposition in the microvasculature, and DIC bleeding [65, 68].

A growing body of evidence reveals that the impact of histones on the above responses is not only related to charge [69] but also mediated through the activation of TLR2 and TLR4 signaling pathways (e.g., ERK, Akt, p38, and NF-*κ*B), the induction of calcium influx, and fibrinogen recruitment [39, 67]. Moreover, histones can increase plasma thrombin generation by reducing protein C activation [70]. Blockading platelets TLR2 and TLR4 with antibodies decrease both the activated platelets and the thrombin generation [39]. Histone-related platelet activation can be prevented in vitro and in vivo by pretreatment with low-dose heparin, which directly antagonizes histones rather than causing anticoagulation [71]. Other therapies also proven to be effective include APC [16], albumin [66], globular C1q receptor (P33) [72], recombinant thrombomodulin (rTM) [68], chondroitin sulfate (CS), and high molecular weight hyaluronan (HMW-HA) associated with the interalpha inhibitor protein (IAIP) [73].

Apparently, a number of the blockers mentioned above, including heparin, albumin, IAIP and HMW-HA, carry negative charges. They can significantly reduce cytotoxicity and platelets activation by neutralizing positive charges and binding with histones. Therefore, we find it reasonable to speculate that negatively charged molecules may naturally have a potent antihistone capacity, which is promising for the development of pharmaceutical drugs to cure histone-related diseases (Table 1).

## 5. Pathologic Roles of Extracellular Histones in ARDS

Despite advances in management over the last several decades, acute respiratory distress syndrome (ARDS) remains an important clinical challenge due to the incomplete understanding of its pathophysiological mechanisms. Recently, a growing body of evidence suggests that extracellular histones contribute to the pathogenesis of ARDS. Histones appear in the bronchoalveolar lavage fluids (BALF) and plasma of patients who developed ARDS after trauma and acid aspiration [18, 19]. By carefully studying these findings, it was determined that the lungs are the most susceptible organ to high levels of circulating histones [18].

### 5.1. Induction of ARDS and Requirements for C5aR/C5L2

Complement component C5a displays the highest inflammatory potency for inducing inflammation [77], which is believed to be involved in the induction of ARDS [42, 78, 79]. As expected, in experimental mice models following airway deposition of LPS or C5a, extracellular histones appear in both the BALF and plasma [42]. However, the presence of extracellular histones is significantly reduced when the mice are knocked out either C5a receptors (C5aR and C5L2) or by the depletion of neutrophils or macrophages [42]. Interestingly, once the histones are present in the extracellular space, cytokine production, epithelial cell damage, barrier dysfunction, and the coagulation cascade activation, which are induced by histones, are independent of C5a receptors [42]. Together, these data indicate that the extracellular histones' appearance requires C5aR/C5L2. However, neutrophil accumulation sometimes occurs with infection without complement activation. It is reported that the absence of C3 or C5 affected neither the accumulation of neutrophils in the lungs nor their appearance in the alveolar space in airway deposition of LPS-induced ALI model [80, 81].

### 5.2. Critical Role for the NLRP3 Inflammasome during ALI

The NLRP3 inflammasome is a multiprotein complex that activates caspase-1, giving rise to the maturation of the proinflammatory cytokines, including IL-1*β* and IL-18, and the induction of pyroptosis [82, 83]. A recent study has suggested an essential role of NLRP3 inflammasome in the development of experimental ALI, as NLRP3 or caspase-1 knock-out mice showed significantly reduced amounts of neutrophil infiltration and albumin leakage in different models of ALI [59]. In addition, extracellular histones can directly activate the NLRP3 inflammasome via the generation of reactive oxygen species as well as the extrusion of K^+  ^ and the elevation of intracellular Ca^2+^ concentration [57–59]. Moreover, NLRP3 and caspase-1 are also required for the presence of extracellular histones during ALI [59], indicating positive feedback and a potential mechanism for inflammatory propagation. Such findings reveal a dynamic interaction between NLRP3 inflammasome and extracellular histones that contributes to ALI.

### 5.3. Barrier Dysfunction and Permeability Changes Induced by Extracellular Histones

**Figure 1:**
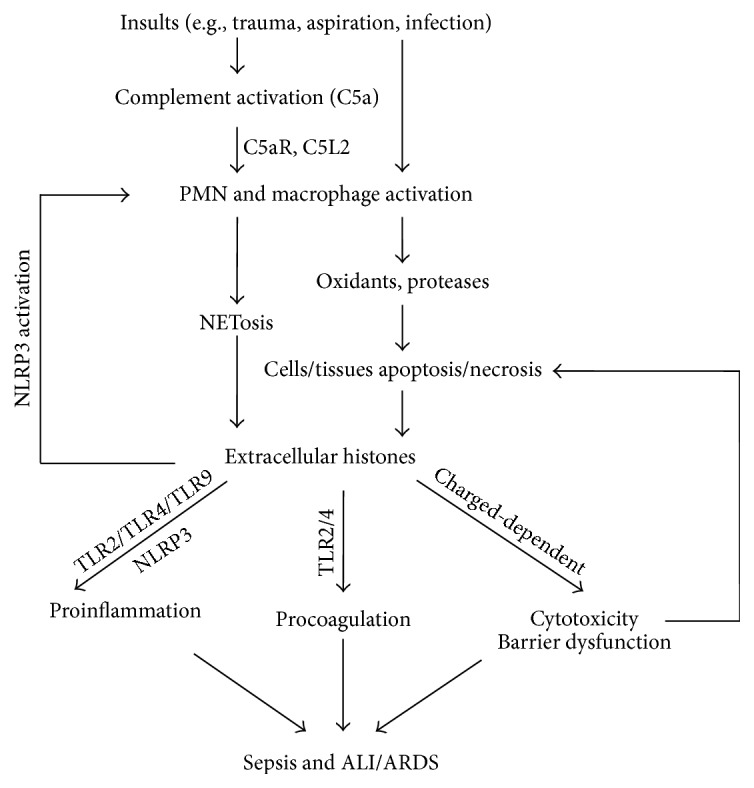
Proposed mechanisms of extracellular histones in the development of sepsis and ALI/ARDS. In response to various physical challenges (e.g., trauma, infection), polymorphonuclear neutrophils (PMN) and macrophages are recruited and activated through complement interaction (C5a and C5a receptors), which is often needed for extracellular histones presented in ALI/ARDS models. However, the accumulation of PMNs sometimes occurs with infection without complement activation. Under these conditions, histones derived from NETosis and dying nonleukocytic cells could be released. Once the histones are present in the extracellular space, they can directly bind to and damage phospholipids in cell membranes in a charged-dependent mechanism, leading to increased membrane permeability and death. They can also act on TLR2, TLR4, and TLR9 and activate the NLRP3 inflammasome to amplify inflammatory responses by the growing release of cytokines and other mediators. Moreover, circulating histones may also enhance coagulation disorders by acting on TLR2 and TLR4. On the other hand, extracellular histones perpetuate detrimental cell/tissue injury and could in turn induce the formation of NETs by activating the NLRP3 inflammasome, which together lead to more histones being released and greater severity of sepsis and ALI/ARDS.

The pathology of ARDS is characterized by an acute inflammatory response linked to the overwhelming recruitment and accumulation of neutrophils, fibrin deposits, alveolar hemorrhage, and pulmonary edema fluid [13, 84, 85]. Recent studies have shown that extracellular histones are responsible for pulmonary edema, which is characterized by increased endothelial and epithelial permeability [18, 42, 86]. In vivo, airway administration of calf thymus histones led to a dose-dependent disruption of the alveolar permeability barrier during ALI, with observations of alveolar albumin leakage and a histological examination revealing obvious lung edema [18, 42]. In vitro, compared with controls, transwells plated with endothelial cells by pretreatment with histones showed that FITC-labeled albumin was significantly elevated in the lower wells, which indicates a histone-induced endothelial permeability increase [18, 86]. Furthermore, recombinant parasite histones also induced endothelial permeability via a charge-dependent mechanism that led to downregulation of the junction protein [86]. Taken together, these data suggest that extracellular histones play a crucial role in barrier dysfunction during ALI. However, in addition to the charge-dependent mechanism, TLR2, TLR4, and TLR9, as the receptors of histones [30, 37, 38], may also give rise to permeability changes, which should be investigated further (Figure 1).

## 6. Clinical Relevance of Plasma and BALF Histones

High concentrations of plasma histones have been detected in patients with sepsis [16, 17] and ARDS [18, 87] and, possibly, correlate with the severity or poor prognosis of these diseases [19, 88]. As observed by Ekaney et al. [17], in septic patients, large amounts of histones are significantly linked to lower endogenous APC levels, a decrease in platelet count, and the need for renal replacement therapy (RRT). Extracellular histones have also been found to predict ICU 28-day mortality in patients with sepsis, and the area under curve (AUC) is 0.744 (*p* = 0.003) with a histone cutoff value of 75 *μ*g/mL (sensitivity 60% and specificity 86.1%) [43]. Moreover, high levels of circulating histones in septic patients are associated with a higher prevalence of new-onset left ventricular dysfunction and arrhythmias (AUC = 0.865, *p* = 0.001 and AUC = 0.813, *p* = 0.001, resp.) [43]. Similarly, in patients with trauma, elevated histone levels are associated with acute lung injury, more days of mechanical ventilation, higher incidences of organ failure, and even higher mortality. An increasing histone level from arrival to 6 h after admission was a multivariate predictor of mortality (hazard ratio 1.005, *p* = 0.013) [88]. In addition, extracellular histones indicate higher mortality in patients with gastric aspiration-induced ARDS [19]. However, extracellular histones are only detectable in 50% of ARDS patients' bronchoalveolar lavage fluid (BALF) from 0 to 10 days after diagnosis. Lower rates of histones are present in BALF samples collected >10 days after diagnosis [42], indicating that histones may only be present in early samples. This might result from treatment with heparin in ICU patients, especially when they receive RRT [17]. Heparin is a highly negatively charged molecule and may bind to positively charged histones to reduce both their cytotoxicity and the number of extracellular histones [89, 90]. Collectively, extracellular histones are significantly elevated in critical diseases, such as sepsis and ARDS, and can reflect severity and mortality, potentially making them a useful and promising biomarker and a therapeutic target.

## 7. Extracellular Histones as Therapeutic Targets

Despite considerable studies into the molecular mechanisms and treatment trials for sepsis and ARDS, the unequivocal and solid curative effect remains limited. However, an increasing body of evidence reveals that histone-related sepsis and ARDS can be inhibited by histone-neutralizing antibodies [27, 30, 38, 91]. For instance, LG2-1 recognizes a peptide from histone H3, LG2-2 reacts with the aminoterminus of H2B, and BWA3 binds to H2A and H4 [92]. More recent studies from Kusano et al. demonstrated that a novel antihistone H1 monoclonal antibody, the SSV monoclonal antibody (SSV mAb), could not merely bind to histone H1 but also exhibited cross-reactivity against histones H3 and H4 [93]. In addition, agonistic activity on TLR2, TLR4, TLR9, and the NLRP3 inflammasome may also provide a potential way to target histones for therapy [30, 37, 38, 57–59]. Moreover, the appearance of extracellular histones requires C5aR/C5L2, and, thus, neutralizing C5a or blockading C5aR/C5L2 may be a potent target that limits the release of histones [42, 76]. As a result of amplifying histone-mediated inflammation through interaction with RAGE and P2Y1 by polyphosphate, targeting polyphosphate, RAGE, and P2Y1 might also have favorable prospects [61]. Moreover, targeting positive charges of histones may be crucial and beneficial because a number of studies have revealed that negatively charged molecules, including heparin [19, 89, 90], albumin [66], C-reactive protein (CRP) [94], endothelial surface protein/gC1q receptor (P33) [72], CS associated IAIP, and HMW-HA [73, 95], could directly bind with histones and abrogate the histone-related pathology. It appears that negatively charged molecules may naturally have a potent antihistone capacity, which is a promising and positive target that needs further investigation. Furthermore, pentraxin 3 (PTX3) also exerts protective effects on sepsis, both in vivo and in vitro, due to its coaggregation with histones [96]. Recombinant thrombomodulin (rTM) could bind to extracellular histones, inhibiting histone-induced platelet aggregation and neutralizing the prothrombotic action of histones [68]. Finally, although FDA-cleared recombinant APC has been withdrawn from the market because of a lack of efficacy in reducing the mortality of sepsis by randomized controlled trials [97], the exact role of APC in hydrolysis and the inactivation of histones has been identified and shows great benefits in a number of experimental studies [16, 30, 86, 98]. Therefore, the appropriate and safe use of APC may still be promising in the early stages of sepsis and ARDS. However, more animal models and clinical randomized controlled trials are needed (Table 2).

## 8. Conclusions and Perspectives

In summary, histones, as the main structure elements, have recently been identified to be present in the extracellular space and to be involved in multiple cellular processes, including cytotoxicity, proinflammation, procoagulation, and barrier dysfunction. Therefore, extracellular histones can help with diagnosis, predict prognosis, and reflect the severity of critical illnesses, including sepsis, ARDS, and septic-ARDS. Antihistone-based therapeutic strategies are thought to be useful and promising. However, there are still many unanswered questions regarding how and when histone-blocking agents should be used and the additive effects of combining different histone-targeted agents. Therefore, the appropriate and safe use of different antihistone-based agents still needs further investigation. Moreover, a better understanding of the substructure, modification modes, and regulation and function of histones in the extracellular space is still needed.

## Figures and Tables

**Table 1:** Sepsis-associated organ dysfunction induced by extracellular histones.

Organ dysfunction	Mechanism	Reference
Lung injury	Cytotoxicity, NLRP3 inflammasome	[42, 59, 74]
Cardiac injury	Cytotoxicity	[43, 60, 74]
Liver injury	Proinflammation	[74, 75]
Kidney injury	Proinflammation, cytotoxicity	[30, 74]
Spleen injury	Cytotoxicity	[74, 76]
Coagulation	Platelets activation, thrombosis	[39, 63, 67]

**Table 2:** Current evidence of targeting extracellular histones for therapy.

Antibody or molecule	Mechanism	References
SSV mAb	Bind to H1; cross-reactivity against H3, H4	[93]
LG2-1	Neutralize H3	[18, 35, 86, 92]
LG2-2	Neutralize H2B	[18, 35, 86, 92]
BWA3	Neutralize H2A and H4	[18, 35, 86, 92]
Anti-TLR2/TLR4/TLR9	Blockade of TLR2/TLR4/TLR9 receptors	[30, 37, 38]
Heparin	Negative charge	[89, 90]
Albumin	Negative charge	[66]
CRP	Negative charge	[94, 99]
SAP	Negative charge	[99]
P33	Negative charge	[72]
IAIP	Negative charge	[73]
HMW-HA	Negative charge	[73, 95]
PTX3	Coaggregation with histones	[96]
rTM	Inhibit histone-induced platelet aggregation	[68]
APC	Degrade histones	[16, 30, 86, 98]

TLR = toll-like receptor, CRP = C-reactive protein, SAP = serum amyloid P component, P33 = endothelial surface protein/gC1q receptor, IAIP = interalpha inhibitor protein, HMW-HA = high molecular weight hyaluronan, PTX3 = pentraxin 3, rTM = recombinant thrombomodulin, and APC = active protein C.
